# Specificity of herbivore‐induced responses in an invasive species, *Alternanthera philoxeroides* (alligator weed)

**DOI:** 10.1002/ece3.3615

**Published:** 2017-11-23

**Authors:** Mu Liu, Fang Zhou, Xiaoyun Pan, Zhijie Zhang, Milton B. Traw, Bo Li

**Affiliations:** ^1^ Institute of Biodiversity Science Ministry of Education Key Laboratory for Biodiversity Science and Ecological Engineering Fudan University Shanghai China; ^2^ Department of Biology Berea College Berea KY USA

**Keywords:** biotic stimuli, coevolution, diet breath, plant invasion, plant–herbivore interactions

## Abstract

Herbivory‐induced responses in plants can both negatively affect subsequently colonizing herbivores and mitigate the effect of herbivory on the host. However, it is still less known whether plants exhibit specific responses to specialist and generalist herbivores in non‐secondary metabolite traits and how specificity to specialists and generalists differs between invasive and native plant populations. We exposed an invasive plant, *Alternanthera philoxeroides*, to *Agasicles hygrophila* (Coleoptera, Chrysomelidae; specialist), *Spodoptera litura* (Lepidoptera, Noctuidae; generalist), manual clipping, or application of exogenous jasmonic acid and examined both the specificity of elicitation in traits of fitness (e.g., aboveground biomass), morphology (e.g., root:shoot ratio), and chemistry (e.g., C/N ratio and lignin), and specificity of effect on the subsequent performance of *A. hygrophila* and *S. litura*. Then, we assessed variation of the specificity between invasive and native populations (USA and Argentina, respectively). The results showed *S. litura* induced higher branching intensity and specific leaf area but lower C/N ratio than *A. hygrophila*, whereas *A. hygrophila* induced higher trichome density than *S. litura*. The negative effect of induction on subsequent larval growth was greater for *S. litura* than for *A. hygrophila*. Invasive populations had a weaker response to *S. litura* than to *A. hygrophila* in triterpenoid saponins and C/N ratio, while native populations responded similarly to these two herbivores. The specific effect on the two herbivores feeding on induced plants did not vary between invasive and native populations. Overall, we demonstrate specificity of elicitation to specialist and generalist herbivores in non‐secondary metabolite traits, and that the generalist is more susceptible to induction than the specialist. Furthermore, chemical responses specific to specialist and generalist herbivores only exist in the invasive populations, consistent with an evolutionary change in specificity in the invasive populations.

## 1 INTRODUCTION

Perceiving and responding to attack by herbivores is an important trait of many plants (Karban & Baldwin, 1997). Induced changes in plants include altered morphological traits (e.g., leaf thickness, Cardenas, Hattenschwiler, Valencia, Argoti, & Dangles, 2015), phytochemicals (e.g., cardenolides, Bingham & Agrawal, 2010), phytohormones (e.g., jasmonic acid, Diezel, von Dahl, Gaquerel, & Baldwin, 2009), and transcription factors (Vogel, Kroymann, & Mitchell‐Olds, 2007). These modifications have been shown to benefit plants by reducing the performance or preference of herbivores attacking the host plants (Agrawal, 2011; Nunez‐Farfan, Fornoni, & Luis Valverde, 2007; Van Zandt & Agrawal, 2004), or mitigating the negative effects of herbivory through tolerance mechanisms, such as compensatory growth, increased photosynthetic rates, or changes in nutrient allocation and uptake (Carmona & Fornoni, 2013; Fornoni, 2011; Stowe, Marquis, Hochwender, & Simms, 2000; Strauss & Agrawal, 1999).

An important question concerning the ecology and evolution of induced plant responses is whether there is specificity to one or various herbivore species (Agrawal, 2001; Strauss, Rudgers, Lau, & Irwin, 2002). There are two important components of specificity (Karban & Baldwin, 1997; Stout, Workman, Bostock, & Duffey, 1998): (1) specificity of elicitation occurs when plants express distinct responses to damage from different herbivores, and (2) specificity of effect occurs when an induced phenotype has different effects on two or more herbivores (Agrawal, 2000; Bingham & Agrawal, 2010; Pashalidou, Lucas‐Barbosa, van Loon, Dicke, & Fatouros, 2013; Van Zandt & Agrawal, 2004).

Previous studies have found consistency on specificity of induced response associated with feeding guilds of herbivores (e.g., chewers versus phloem‐feeders, see review in Ali & Agrawal, 2012). Furthermore, many studies have found that herbivores with distinct diet breadth (specialization) can still influence plant responses differentially (Agrawal, 2000; Van Zandt & Agrawal, 2004; Vogel et al., 2007). However, the systematic review suggests that there is still no consistent pattern of differential elicitation based on the degree of host plant specialization (Ali & Agrawal, 2012). One of reasons for this inconsistent pattern may be that most studies have concentrated more on response of plant secondary metabolites than on other plant traits (e.g., physical defense traits, Barton, 2016). Nevertheless, a meta‐analysis of 72 studies suggests that although plant secondary chemicals are seen as fundamental to the defense against insects, some nonsecondary metabolite plant traits (life‐history traits, physical defense traits, gross morphological traits, and primary chemistry) can also play an important role in plant defense against herbivores (Carmona, Lajeunesse, & Johnson, 2011). In particular, Carmona et al. (2011) have found that the negative effects of physical defense (e.g., trichome density and leaf toughness) and life‐history traits (e.g., the rate of growth and phenology) are stronger than those of secondary metabolic chemistry on specialist herbivores. Thus, it is necessary to assess specificity of elicitation to herbivores with different diet breadths in a wide range of plant traits, especially in traits other than secondary metabolites.

Moreover, despite substantial evidence for specificity of induced response, it is still little known how such specificity could evolve by natural selection (Agrawal, 2011). Some recent studies have indicated that invasive populations of exotic plants may have different specificity of induced response to specialist and generalist herbivores relative to native populations (Huang et al., 2010; Wang et al., 2013). However, the patterns remain highly controversial (Huang et al., 2010; Wang et al., 2012, 2013). Wang et al. (2013) found that specificity of extrafloral nectar (EFN) induction to specialists vs. generalists only exists in native *Triadica* populations rather than invasive ones, whereas Huang et al. (2010) found the opposite in tolerance responses of *Triadica*. Therefore, limited traits and plant species investigated restrict broader generalization regarding how invasive and native populations vary in specificity to sets of herbivores with different diet breadth.

In this study, we assessed specificity of induced response in a wide range of plant traits (fitness, morphology, and chemistry) and the potential differences of populations in the alligator weed, *A. philoxeroides*, which is native to South America, but has colonized large regions of the United States, Australia, and China (Julien, Skarratt, & Maywald, 1995). Here, we compared invasive populations from the United States with native populations from Argentina to understand how plants from these regions respond to the attack by both *A. hygrophila* (Colepotera: Chrysomelidae), a specialist from South America and *S. litura* (Lepidoptera: Noctuidae), a generalist from Asia. To better understand the effects of herbivore induction, we followed the recommendations of Ali and Agrawal (2012) and included a manual clipping treatment and an exogenous jasmonic acid (JA) application treatment. We therefore compared both types of induction treatments relative to undamaged plants and two abiotic stimuli: manual clipping and exogenous jasmonic acid (JA). Specifically, we asked:

1. Is there any specificity of induced response in plant traits other than secondary metabolites?
2. What is the difference in specificity of induced response between invasive and native populations of *A. philoxeroides*?

## 2 MATERIALS AND METHODS

### 2.1 Study species

**Figure 1:**
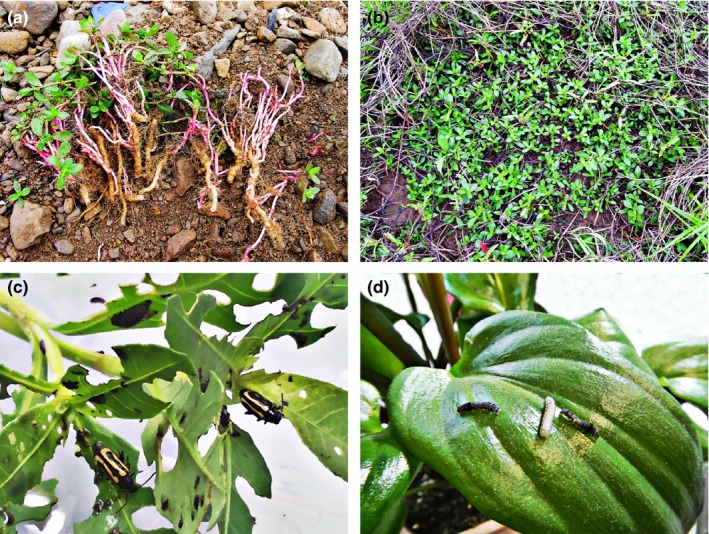
(a) *Alternanthera philoxeroides* reproduces from storage roots, (b) a dense monospecific stand of *A. philoxeroides*, (c) a second‐instar larva and two adults of *Agasicles hygrophila* feeding on *A. philoxeroides*, (d) three third‐instar larva of *Spodoptera litura* feeding on *Drimiopsis kirkii*

*Alternanthera philoxeroides* (alligator weed) is a perennial herb (Amaranthaceae), which depends on storage roots and rhizomes to overwinter (Jia, Pan, Li, Chen, & Yang, 2009). The plant reproduces primarily through vegetative propagation and emerges from storage roots in spring to form dense monospecific stands (Figure 1a,b; Jia, Pan, Sosa, Li, & Chen, 2010). The native range of this species extends from Argentina (39°S) to southern Brazil (18°S), where it is attacked by as many as 40 insect herbivores, including specialists like the alligator weed thrip, *Amynothrips andersoni*, alligator flea beetle, *A. hygrophila*, and a caterpillar, *Arcola malloi* (Maddox, Andres, Hennessey, Blackburn, & Spencer, 1971), but in its invasive range, which includes both the United States and China, few insects feed on alien *A. philoxeroides* (Xiaoyun P., personal observation).

*Agasicles hygrophila* (Coleoptera: Chrysomelidea, Figure 1c) is a strictly specialized leaf‐chewing insect that completes its entire life cycle on alligator weed and has a distribution including all of the native alligator weed range. It has been utilized as biological control agent in USA since 1964 and in China since 1986. Despite these efforts, the insect is absent from many invasive populations of alligator weed, especially in terrestrial habitats (Appendix S1; Coulson, 1977; Ma, 2001; Pan, Zhang, Dong, & Li, 2010).

*Spodoptera litura* (Lepidoptera: Noctuidae, Figure 1d) is a cosmopolitan chewing insect that feeds on approximately 150 plant species from 40 families including plants in the Amaranthaceae (Rao, Wightman, & Rao, 1993).The native distribution of *S. litura* includes most of Asia, Australia and extends into the south Pacific as far west as Hawaii, but it is not found in North America (Nagoshi, Brambila, & Meagher, 2011; Pogue, 2002).

In this study, we obtained stem fragments from five invasive USA populations and five native Argentine populations in 2005 and 2006, respectively (Appendix S1), and planted them in the glasshouse. We collected 80 adults of *A. hygrophila* locally and maintained the colony on Chinese *A. philoxeroides* prior to the experiment. Those collected insects were fed in a growth chamber for 1 month (one generation) before experiment to reduce possible maternal effects (influence of heterogeneity of parental phenotype on their offspring phenotype). We purchased *S. litura* from the KeYun biocontrol company in Henan province, China, and maintained the colony on artificial diet (main nutrient content are wheat germ [12%], sugar [2%], casein [4.5%], agar [1.5%], Wesson's salt [1.0%], sorbic acid [0.4%], and ascorbic acid [0.5%]). we collected over 120 stem fragments from each of 10 populations (five invasive and five native populations) to propagate seedlings.

### 2.2 Common garden experiment

We used a completely randomized design (CRD) in the experiment. On 28 August 2015, 60 seedlings from each population with similar leaf numbers (two pair of leaves) were chosen and transferred to 400 ml plastic pots with fertile soil (Beilei Organic Fertilizer Co., Ltd., Zhenjiang, China) with the content of N, P, K ≈ 2 ± 0.3% (untransformed mean ± *SD* and the same below, dry weight basis), organic matter ≈35 ± 5% (dry weight basis), water ≈45 ± 3%, and pH = 6 ± 0.5 (total *n* = 600). Those 60 seedlings of each populations were averagely divided into four replicated plots (150 plants per plot). We randomly arranged the pots in one plot and caged each individual plant over the course of the experiment to prevent disturbance from other insects in the glasshouse.

On September 21, we randomly assigned one‐fifth of the plants (*n* = 3) of each population in each plot to one of five treatments (unmanipulated control, *A. hygrophila* herbivory induction, *S. litura* herbivory induction, manual clipping, and exogenous JA) and each population/treatment combination was replicated four times (four plots). For herbivory induction treatments, we applied two second‐instar *A. hygrophila* or two third‐instar *S. litura* larvae on each individual plant. A preliminary observation suggested that these instars would provide the equivalent amount of damage (50 ± 10% leaf area damage after 12 hr). Those two larvae were, respectively, confined on two fully expanded new leaves, which represented 25% leaf area (eight leaves totally in each plant, Appendices S2 and S3). We removed larvae when the two caged leaves were consumed completely (for 2d). For the manual clipping treatment, we clipped two leaves over 2d (one leaf per day) by cutting the whole leaf at the base of the stem (Appendix S2). For the exogenous jasmonic acid treatment, we applied 1 mmol/L of jasmonic acid solution (adding 1 ml of EtOH [95%] to 100 mg of pure jasmonic acid [Sigma J250001] and then diluting 100 μl of JA solution into 50 ml of pure water) onto the upper surface of each leaf with a paintbrush (Appendix S2, A preliminary experiment found that *A. philoxeroides* began to exhibit significantly induced response at 1 mmol/L [the concentration gradient was 1 mmol/L, 2.5 mmol/L, 5 mmol/L, and 7.5 mmol/L]).

To avoid cross‐interference by herbivore‐induced volatiles (HI‐VOCs), we grouped 120 plants of the same treatment at the beginning of induction and we: (1) kept about 1.5 m of distance between each treatment group with its neighbors (the effective radius of volatiles can be range from 60 cm to 1 m [Heil, 2014]), (2) assigned the control treatment to be upwind of other treatment groups, and (3) made the JA induction at an equivalent, adjacent glasshouse module (these two adjacent glasshouse modules were the same size [10 × 6 × 8 m, length × width × height, QiuShi phytotron company, ZheJiang Province, China] and were regulated to the same climate conditions [26 ± 3°C, 60 ± 5% RH, 16 hr:8 hr, day: night illumination cycle]). We re‐randomized plants again after 2 days when treatments were finished because these volatiles can be released immediately following damage and the release can cease rapidly after damage stops (usually within several minutes [Arimura, Shiojiri, & Karban, 2010]).

### 2.3 Larval bioassay

Seven days after induction treatments, 12 plants of each population/treatment combination were divided into three groups (10 populations × 5 treatments × 4 replicates = 200 plants per group). Two groups were challenged by either three second‐instar *A. hygrophila* or third‐instar *S. litura* larvae, and the remaining group was kept for phenotypic measurements. We retained about 1.5 m of distance between each bioassay group and its neighbors to prevent crosstalk. The bioassay larvae were weighed to 0.0001 g initially and allowed to feed freely in nylon mesh bags for 4 days, after which the larvae were removed and reweighed by electronic balance (FA2104, Shanghai, China). We used the difference between post‐ and prelarval biomass as our measurement of larval growth gain in the challenge bioassay.

### 2.4 Plant measurements

On 9 October 2015, the plants in the group not challenged by herbivores were harvested to measure plant fitness, morphological, and chemical traits. We measured leaf areas with a leaf area meter (Li‐3100, Li‐Cor Inc., Lincoln, NB, USA) and determined leaf trichome numbers by removing a 0.5‐cm‐diameter leaf disk from the first fully expanded leaves and counting the total number of trichomes on the top and bottom surfaces. All plant material was separated into below and aboveground (leaves, branches, and stems) parts, and dried at 60°C for 72 hr prior to determination of dry biomass. We used plant total, aboveground, and belowground biomass as our estimates of plant fitness (fitness traits). Our morphological traits included trichome density (sum of top and bottom trichome number on the disk), root:shoot ratio (RSR; ratio of belowground biomass and aboveground biomass), branch intensity (BI; ratio of branch dry weight and stem dry weight), specific stem length (SSL; ratio of length of stem in cm and stem dry weight in g), and specific leaf area (SLA; ratio of leaf area in cm^2^ and leaf dry weight in g).

To obtain sufficient material for chemical analyses, we pooled dry leaves of four replicate individuals within each population/treatment combination and ground them to a fine powder. To measure total triterpenoid saponins, we used UV spectrophotometry (Wang, Xu, & Wang, 2011). Leaf powder was weighed, soaked in diethyl ether, heated in a water bath at 30°C for 4 hr, and then centrifuged. The sediment was extracted by methyl alcohol and chloroform, successively. The chloroform was desiccated, and the extract was dissolved in 200 μl methyl alcohol. The solution was measured at 215 nm, and the concentrations was calculated by regression, using oleanolic acid as a standard. To measure total flavonoids, we also used UV spectrophotometry (Li, Zhang, Xu, Wang, & Zhang, 2017; Zhang, Li, He, Chen, & Liu, 2012). The leaf powder was weighed, soaked in 70% ethyl alcohol, and then centrifuged. The supernatant was collected, desiccated to remove the ethyl alcohol, and further extracted in distilled water and 1 ml of 99% ethyl acetate. The ethyl acetate was removed by desiccation, and the extract was dissolved in 1 ml of 70% ethyl alcohol with 1 ml KOH. After 5 min, the solution was diluted to 5 ml using 70% ethyl alcohol. The absorbance of the extract was measured at 395 nm, and concentrations were calculated by regression, using luteolin as a standard. To determine the lignin concentrations, we used the Klason method (Effland, 1977), mixing equivalent leaves of each population of parent material together and using the Klason lignin and absorbance by UV spectrophotometry at 280 nm for each combination of population and treatment (Johnson, Moore, & Zank, 1961). To determine total carbon, total nitrogen, and the C/N ratio, we used an elemental autoanalyzer (FlashEA 1112 Series, Thermo Inc., Milan, Italy).

### 2.5 Statistical analyses

We used mixed‐model ANOVAs (SPSS Proc GLM; v 19.0, SPSS Institute Inc, 2010) to test effects of treatments (control, *A. hygrophila* damage, *S. litura* damage, clipping, JA), origin (native versus invasive), and their interactions on plant fitness traits, morphological traits, and chemical traits, and insect growth gain in the challenge bioassay. We used the Anderson‐Darling test and Levene's test to assess normality and homoscedasticity of the residuals from the ANOVAs. To achieve normality and homoscedasticity of residuals, we used log transformation (total biomass, aboveground biomass, belowground biomass, RSR, SSL, and total trichome density), reciprocal transformation (SLA), and Box‐Cox transformation (BI). In the cases where transformations were performed, the results are presented as back‐transformed means and standard errors. Least significant difference (LSD) post hoc tests were used to contrast specific means. For the herbivore bioassay, each individual plant could only be fed upon by one kind of herbivore, so we used population averages for the LSD post hoc test rather than data at the level of individual plants. We treated population nested within origin as a random effect.

## 3 RESULTS

### 3.1 Plant fitness traits

**Figure 2:**
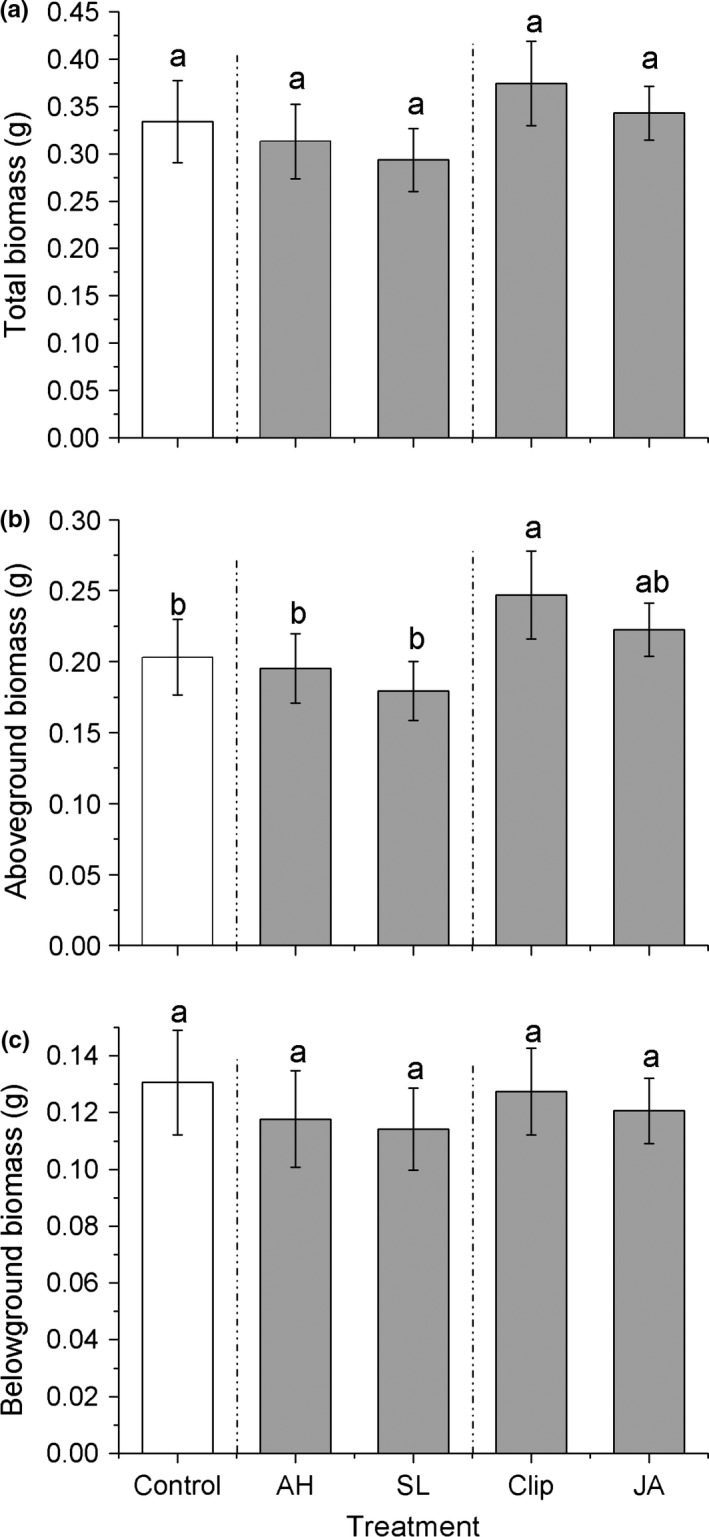
Effects of the induction treatments on plant fitness traits, (a) total plant biomass, (b) aboveground biomass, (c) belowground biomass among *Alternanthera philoxeroides* plants. AH*, Agasicles hygrophila* damage, SL, *Spodoptera litura* damage, Clip, clipped leaves, JA, exogenous jasmonic acid application. Data are means ± 1 *SE*, and different letters indicate significant differences among means following LSD‐adjusted post hoc contrasts

The type of treatment had a significant effect on plant aboveground biomass (Table 1). Clipping treatment plants had 25% greater aboveground biomass than controls and 43% greater aboveground biomass than feeding damage plants (Figure 2b), but similar with JA. Treatments had no significant effects on plant total biomass or belowground biomass (Table 1, Figure 2a,c).

**Table 1:** The mixed‐model ANOVA tests the effect of induction treatment, continental origin (native vs. invasive), their interaction on three square‐root transformed fitness traits (total biomass, aboveground biomass, and belowground biomass). Population (Origin) of *Alternanthera philoxeroides* was treated as a random factor. Statistical significance is marked in bold and indicated as: *p < .05, **p < .01, ***p < .001

Source	*df*		*F* ratio		
			Total biomass	Aboveground biomass	Belowground biomass
Treatment	4	182	1.53	2.39*	0.52
Origin	1	8	0.4	0.02	2.37
Population(Origin)	8	182	11.06***	11.37***	10.27***
Origin × treatment	4	182	1.19	1.11	1.12

### 3.2 Plant morphological traits

**Figure 3:**
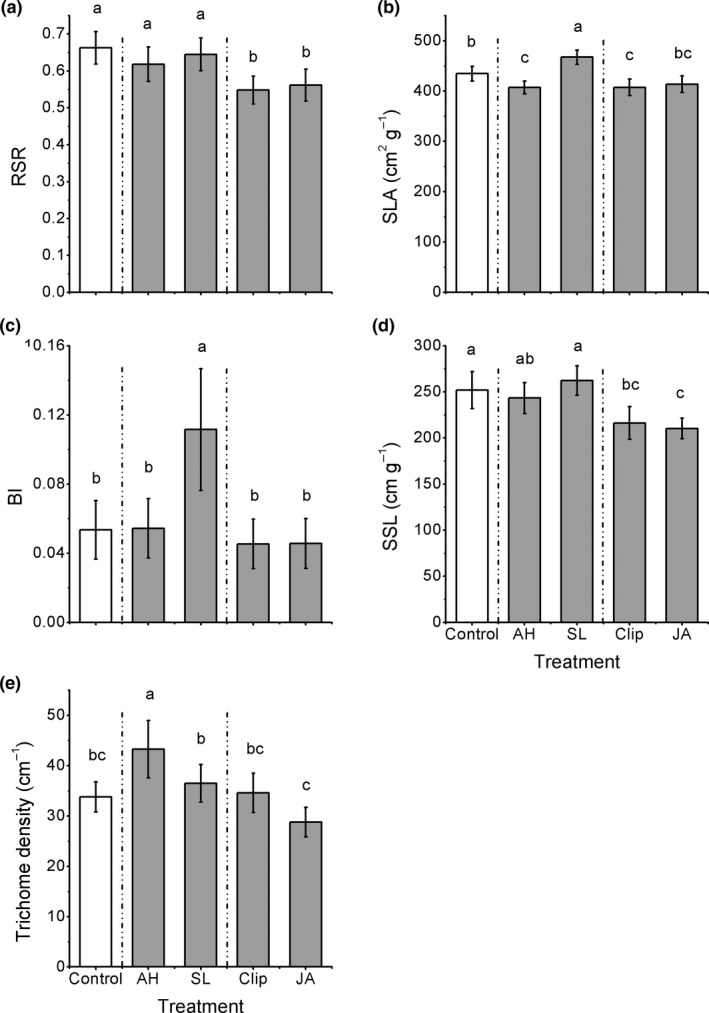
Effect of the induction treatments on morphological traits of *Alternanthera philoxeroides*, including (a) root:shoot ratio (RSR), (b) branching intensity (BI), (c) specific leaf area (SLA), (d) specific stem length (SSL), and (e) trichome density. AH*, Agasicles hygrophila* damage, SL*, Spodoptera litura* damage, Clip, clipped leaves, JA, exogenous jasmonic acid. Data are means ± 1 *SE*, and different letters indicate significant differences among means following LSD‐adjusted post hoc contrasts

The treatments had significant effects on all morphological traits (Table 2). Compared to controls, plants induced by the *S. litura* treatment significantly increased branching intensity, BI (+120%) and specific leaf area, SLA (+7%), while plants induced by the *A. hygrophila* treatment did not change BI, but −7% decreased SLA (Figure 3b,c). In addition, for trichome density, plants in the *S. litura* treatment were similar with controls, but *A. hygrophila* treatment plants had higher densities than did in control plants (+26%) (Figure 3e).

**Table 2:** The mixed‐model ANOVA tests the effect of induction treatment, continental origin (native vs. invasive), their interaction on five morphological traits (root:shoot ratio, RSR, Box‐Cox transformed branch index, BI, reciprocal‐specific leaf area, SLA, specific stem length, SSL, square‐root total trichome density). Population (Origin) of *Alternanthera philoxeroides* was treated as a random factor. Statistical significance is marked in bold and indicated as: *p < .05, **p < .01, ***p < .001

Source	*df*		*F* ratio				
			RSR	BI	SLA	SSL	Trichome density
Treatment	4	182	4.45**	3.895**	7.254***	3.80**	2.36*
Origin	1	8	13.81**	10.520*	1.199	1.88	0.61
Population(Origin)	8	182	6.63***	7.415***	8.783***	11.68***	11.99***
Origin × treatment	4	182	0.04	1.874	0.573	1.27	1.27

Compared to plants induced by abiotic stimuli, plants in the two biotic treatments had higher root:shoot ratio, RSR (Figure 3a). Plants induced by the *S. litura* damage had higher BI (+140% on average) and SLA (+14% on average), but plants in the *A. hygrophila* treatment did not change those traits (Figure 3b,c). For specific stem length, SSL, plants induced by *S. litura* treatment were higher than plants in the two abiotic treatments (+23% on average), while *A. hygrophila* treatment plants were only higher than plants in the JA treatment and did not differ from clipped plants (Figure 3d). For trichome density, plants induced by *S. litura* treatment were only higher than JA treatment plants (+29%), but similar to the clipped plants, while plants induced by *A. hygrophila* treatment were higher than both abiotic treatments (+38% on average, Figure 3e).

When comparing the two biotic treatments with each other, plants induced by *S. litura* had significantly higher SLA and BI than plants in *A. hygrophila* treatment (8.6% and 106%, respectively, on average, Figure 3b,c), although *A. hygrophila* induced more of an increase in trichome density than *S. litura* did (19% on average, Figure 3e).

### 3.3 Plant chemical traits

**Figure 4:**
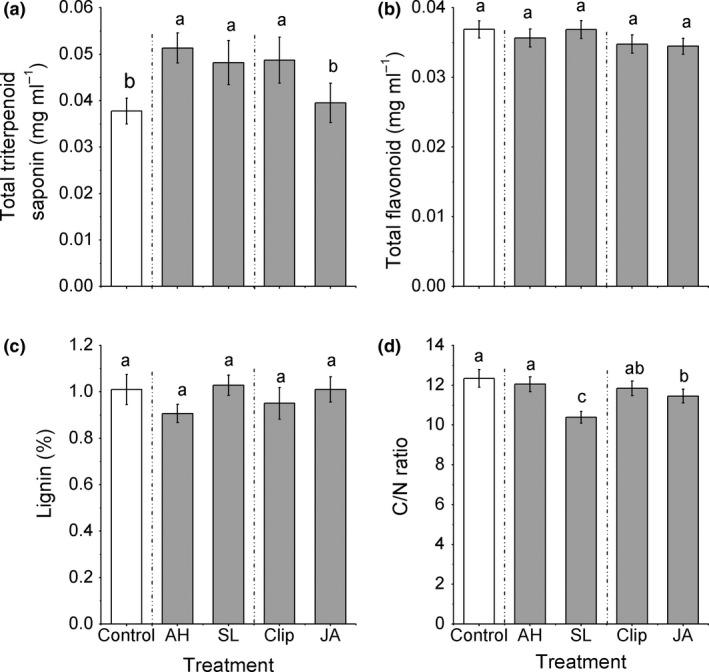
Effect of the induction treatments on chemical traits of *Alternanthera philoxeroides*, including (a) total triterpenoid saponins, (b) total flavonoid, (c) lignin concentrations, (d) C/N ratio. AH*, Agasicles hygrophila* damage, *Spodoptera litura,* SL, damage, Clip, clipped leaves, JA, exogenous jasmonic acid. Data are means ± 1 *SE*, and different letters indicate significant differences among means following LSD‐adjusted post hoc contrasts

The treatments had significant effects on total triterpenoid saponin concentrations and C/N ratio (Table 3). Compared to controls, the two herbivory and clipping treatments had significantly higher total triterpenoid saponins (35.9%, 27.6%, 29.0% on average, respectively), while JA induction had similar concentrations (Figure 4a). Meanwhile, only the *S. litura* and JA treatments resulted in a significantly decreased C/N ratio (−15.8% and −7.2% on average, respectively, 12.34 ± 1.47 [control]; 12.05 ± 1.27 [*A. hygrophila*]; 10.39 ± 0.98 [*S. litura*]; 11.84 ± 1.24 [clip]; 11.45 ± 1.16 [JA], Figure 4d).

**Table 3:** The mixed‐model ANOVA tests the effect of induction treatment, continental origin (native vs. invasive), and their interaction on four plant chemical traits (total triterpenoid saponin, total flavonoid, lignin, carbon nitrogen ratio, C/N ratio). Population (Origin) of *Alternanthera philoxeroides* was treated as a random factor. Statistical significance is marked in bold and indicated as: *p < .05, **p < .01, ***p < .001

Source	*df*		*F* ratio			
			Total triterpenoid saponin	Total flavonoid	Lignin	C/N ratio
Treatment	4	32	6.44**	2.002	0.878	14.460**
Origin	1	8	23.332**	0.293	0.178	1.001
Population(origin)	8	32	2.201	10.384***	1.266	14.344***
Origin × treatment	4	32	11.426***	1.547	2.316	2.877*

Compared to the two abiotic stimuli, plants induced by the two herbivores did not differ from the clipped plants in total triterpenoid saponins concentrations, but plants induced by the *S. litura* and *A. hygrophila* exhibited triterpenoid saponins concentrations that were 30.0% and 22.0% higher than the JA treatment on average, respectively (Figure 4a). Plants induced by the *A. hygrophila* did not differ in C/N ratios from clipped plants, but were higher than plants treated with exogenous JA (5.2% on average, Figure 4d), while plants induced with the *S. litura* had lower C/N ratios relative to the two abiotic treatments (12.3% and 9.3% on average for clipping and exogenous JA, respectively, Figure 4a). For total flavonoids and lignin, there were no significant differences among treatments (Table 3, Figure 4b,c).

In addition, although the two biotic stimuli did not differ in their effects on total triterpenoid saponins concentrations, the *S. litura* treatment decreased C/N ratios significantly relative to the *A. hygrophila* treatment (16.0% on average, Figure 4d). In contrast, the clipped plants had higher total triterpenoid saponins concentrations than those induced with JA (23.3% on average, Figure 4a), but did not differ in C/N ratio.

### 3.4 Effect on insect performance

**Figure 5:**
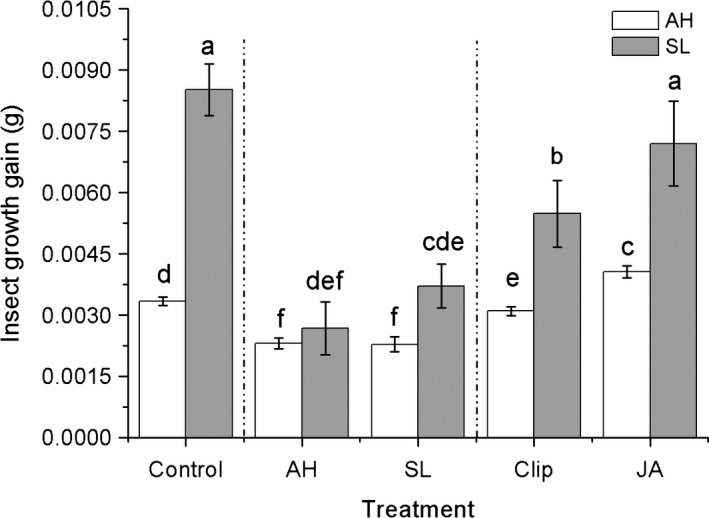
Comparison of two larval growth gain (*Agasicles hygrophila*, white bar; *Spodoptera litura*, black bar) for each of the five treatments. AH*, A. hygrophila* damage, SL*, S. litura* damage, Clip, clipped leaves, JA, exogenous jasmonic acid. Data are means ± 1 *SE*. different letters indicate means that differ significantly following LSD‐adjusted post hoc contrasts

The specificity of effect can be detected from significant interactive effect between treatment and bioassay (Table 4). Compared to growth gain of *S. litura* in control, the *S. litura* experienced a significantly decreased growth gain in the two herbivory and clipping treatments (−69%, −56%, and −36% on average, respectively, relative to control, Figure 5), while not affected by JA. Additionally, the *A. hygrophila* was also negatively affected by the two herbivory damage and clipping induction (−31% and −32% and −7%, respectively, on average relative to the control,) but positive affected by JA (22% on average, Figure 5).

**Table 4:** The mixed‐model ANOVA tests the effect of induction treatment, continental origin (native vs. invasive), their interaction on insect performance (insect growth gain after feeding on each treatment plants). Analyses are separated by specific bioassay insect. Population (Origin) of *Alternanthera philoxeroides* was treated as a random factor. Statistical significance is marked in bold and indicated as: *p < .05, **p < .01, ***p < .001

Source	*df*		*F* ratio
Treatment	**4**	**355**	23.683***
Bioassay	1	355	80.365***
Origin	1	355	6.899*
Population	8	355	1.823
Treatment × bioassay	**4**	**355**	8.081***
Treatment × origin	4	355	1.663
Bioassay × origin	**1**	**355**	7.592***
Treatment × bioassay × origin	4	355	1.418

### 3.5 Variation in specificity between native and invasive populations

For most plant traits, the treatments did not differ between the native and invasive populations (no significant interactive effect of treatment and origin in Tables 1, 2, 3). Nevertheless, there was a significant interaction between origin and induction treatment on total triterpenoid saponins concentration and C/N ratio (Tables 2 and 3).

**Figure 6:**
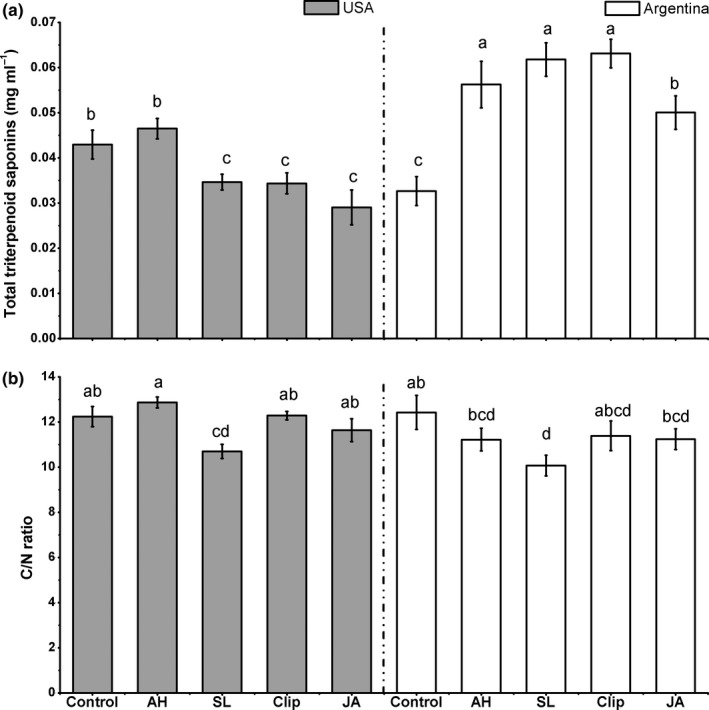
The influence of continental origin of populations and five treatment on (a) total triterpenoid saponins, (b) C/N ratio. AH, *Agasicles hygrophila* damage, SL, *Spodoptera litura* damage, Clip, clipped leaves, JA, exogenous jasmonic acid. Data are means ± 1 *SE*, and different letters indicate significant differences among means following LSD‐adjusted post hoc contrasts

Compared to the control, the invasive populations had lower total triterpenoid saponins concentrations in the *S. litura* treatment (−19% on average) and two abiotic treatments (−20% for clipping and −30% for JA on average) but no significant induced changes in the *A. hygrophila* treatment (Figure 6a). Nevertheless, native populations significantly increased triterpenoid saponins concentrations to all the induction treatments compared to controls (70%, 88%, 91%, and 52% on average, respectively) (Figure 6a). The invasive populations had lower total triterpenoid saponin contents in the *S. litura* treatment relative to the *A. hygrophila* treatment (−26%; Figure 6a), while native populations responded with similar triterpenoid saponin contents to the two biotic treatments (Figure 6a). Invasive populations damaged by *A. hygrophila* had higher total triterpenoid saponins than either of the two abiotic treatments (38% for clipping and 62% for JA on average, respectively; Figure 6a), while there were no significant differences between *S. litura* and the two abiotic treatments. In native populations, the two biotic treatments had similar total triterpenoid saponins with clipping treatment but significantly higher total triterpenoid saponins than the JA treatment (12% for *A. hygrophila* and 24% for *S. litura* on average, respectively; Figure 6a).

Both invasive and native populations had significantly decreased C/N ratios following *S. litura* damage (−13% and −19% on average, respectively) but no significant induced changes in the other three induction treatments compared to controls (Figure 6b). The invasive populations damaged by *S. litura* had lower C/N ratio than *A. hygrophila* treatment (−17% on average), while native populations showed similar response in C/N ratio to the two herbivores (Figure 6b). Invasive populations had significant lower C/N ratio after damage by *S. litura* compared with the two abiotic treatments (−13% for clipping and −8% for JA on average, respectively), but there was no significant difference between the *A. hygrophila* treatment and the two abiotic treatments. In native populations, there were no significant differences between biotic and abiotic treatments.

Additionally, the population origin did not influence the magnitude of growth gain differences between the two herbivores in response to the treatments (no significant interactive effect among treatment, bioassay, and origin in Table 4).

## 4 DISCUSSION

### 4.1 Specificity of elicitation in different plant traits and specificity of effect

In this study, we found that these plants can express specific elicitation to *S. litura* and *A. hygrophila* in some traits. In detail, the *S. litura* induced significantly decreased C/N ratio relative to the *A. hygrophila* and other treatments in our study. This C/N ratio response is consistent with a previous finding that generalist swift moth *Endoclita excrescence* decreases the foliage C/N ratio of willow species (Utsumi & Ohgushi, 2009) and indicates that plants can express specific elicitation to herbivory in C/N ratio. However, few studies have concentrated on the specificity of elicitation in C/N ratio to feeding by two herbivores that differ in diet breadth (Mooney, Tiedeken, Muth, & Niesenbaum, 2009; Wang et al., 2012), and the responses of C/N ratio in our experiment are in contrast to those studies. Mooney et al. (2009) have found that the C/N ratio of *Lindera benzoin* has no significant difference in response to herbivory from specialist, *Epimecis hortaria*, and generalist, *Spodoptera exigua*, compared to controls, while Wang et al. (2012) have found *Triadica sebifera* tends to similarly increase C/N ratio in response to specialist, *Gadirtha inexacta*, and two generalists, *Grammodes geometrica* and *Cnidocampa flavescens*. The reason underlying the differential response of C/N ratio in different research systems may be that the defenses of the herbaceous plants which evolved in fertile soils on disturbed sites like *A. philoxeroides* are generally nitrogen rather than carbon based and have higher tolerance to herbivory relative to more slowly growing woody plants (Bryant, Chapin, & Klein, 1983; Niesenbaum, 1992; Zhang, Pan, Zhang, He, & Li, 2015). Therefore, the C/N ratio response may vary in different types of plant growth form. However, it is still unclear whether the C/N ratio response to different herbivory depends on the strategies of resource allocation between plant defense and growth, and it is necessary to test the specificity of elicitation in C/N ratio to generalist and specialist in diverse research systems.

Our finding also showed that *A. philoxeroides* exhibited significantly higher trichome density following damaged by the specialist *A. hygrophila* than when damaged by generalist *S. litura*. It is consistent with an emerged finding that plants can express specific elicitation in trichome to different herbivores especially with distinct diet breadth (Traw & Dawson, 2002).

Additionally, our results show that the induced plant responses to biotic stimuli were different from the responses to abiotic stimuli in fitness (e.g., aboveground biomass), chemical (e.g., concentrations of triterpenoid saponin and C/N ratio), and some morphological traits (e.g., SLA and trichome density). These results indicate that most plant traits have some specificity of elicitation to biotic and abiotic stimuli although with some overlap, consistent with previous observations (Mooney et al., 2009; Travers‐Martin & Mueller, 2008). The specific responses to biotic and abiotic stimuli may be based on two facts: (1) plants have recognition of herbivore‐specific cues such as herbivore saliva (Heil, 2009; Hilker & Meiners, 2010) and (2) clipping is usually an one‐off wounding event, while real herbivores normally cause a longer time of damage to plant tissue, which could increase the intensity of induction (Moreira, Zas, & Sampedro, 2012).

In this study, we also found that no matter what the induction treatment is, the generalist *S. litura* experienced more decrease of growth gain on induced plants relative to control than did specialist *A. hygrophila*. (Figure 4). Considering that *S. litura* is a typical generalist herbivore and *A. hygrophila* is a strictly specialist (see Section 2), that result may support the emerged view that generalists are more susceptible to induced plant defense than are specialist herbivores (Coley, Bateman, & Kursar, 2006; Cornell & Hawkins, 2003; Van Zandt & Agrawal, 2004). However, more recent studies indicate that the relative susceptibility between generalist and specialist herbivores is correlated with specific plant traits. For example, a recent meta‐analysis found that physical and life‐history plant traits are both negatively correlated with susceptability of specialist herbivores, while there was no consistent relationship between those traits and susceptibility of generalist herbivores (Carmona et al., 2011).

To rule out the chance that these differences in response are due to a difference in the amount of damage per se, we carefully standardized the amount of damage that the two herbivores applied to plants (cages were used to limit each herbivore on a specific leaf [Appendix S3] and just two leaves of each plant were eaten completely. These two leaves were 25% of total leaf area). Also, the observed differences were not caused by different feeding guild of herbivores, because the two kinds of larvae are both chewing feeders and have similar feeding behaviors, in that both herbivores cut the leaf from margin of leaf to midrib (Appendix S4).

### 4.2 Variation of specificity between native and invasive plant populations

For the comparison of elicitation to the two herbivores, the invasive populations expressed lower total triterpenoid saponins and C/N ratio to generalist *S. litura* relative to specialist *A. hygrophila,* whereas native populations had no such specificity to the two herbivores (Figure 6). These results support the view that invasive and native populations differ in their specificity of elicitation to herbivores with distinct diet breadth (Huang et al., 2010; Wang et al., 2013). Furthermore, these results indicate that specificity of elicitation only existed in invasive *A. philoxeroides* populations rather than in native populations. This finding is consistent with previous research that invasive *Triadica sebifera* populations can express specific tolerance to generalist and specialist herbivores, while native populations did not have this specificity (Huang et al., 2010). However, in contrast, other important research on indirect defense (extrafloral nectar, EFN) indicates loss of specificity to generalist and specialist herbivores in invasive populations (Wang et al., 2013). Those conflicting results suggest that reduced herbivory may not lead to a consistent variation in specificity of induced response between invasive and native populations; rather variation in specificity between two populations may be correlated with the function of specific plant traits (Wang et al., 2013). Nevertheless, further research is still needed to assess the variation in specificity to herbivores with different diet breadth between invasive and native populations in diverse research systems.

For comparison of specific response to biotic and abiotic treatments, there is different specificity between invasive and native populations. This result is consistent with previous findings in *Boechera stricta* that the specificity of elicitation to biotic and abiotic stimuli exists in some genotypes but not in others (Manzaneda, Prasad, & Mitchell‐Olds, 2010). Besides, in our study, the specificity of elicitation to biotic and abiotic stimuli in invasive populations also varied between types of biotic stimuli. In other words, specificity only existed between either of *A. hygrophila* or *S. litura* treatment and abiotic treatments in invasive populations. However, in native populations, the specific elicitation to biotic and abiotic treatments did not vary with different biotic stimuli (Figure 6).

Additionally, in this study, we did not detect variation in specificity of effect between invasive and native populations. In other words, no matter where host plants were from, the generalist *S. litura* was always more susceptible to the particular induced phenotype than the specialist *A. hygrophila*. This finding is consistent with previous research that there is no variation in specificity of effect on three specialist herbivores among different genotypes of *Solidago altissima* (Uesugi, Poelman, & Kessler, 2013).

Finally, it is also worth noting that if ignoring variation of invasive and native populations, the induced response of triterpenoid saponins has no specificity to the two herbivores (Figure 4a). This result suggests that a lack of distinguishing plant population variation will cover up the specificity of plant induced response (Uesugi et al., 2013).

Overall, our study shows strong evidence for both specificity of elicitation in a wide range of traits, especially in traits other than secondary metabolites and specificity of effect. Furthermore, our data demonstrate that invasive populations can express specificity of elicitation to different herbivores, but native populations cannot. We also suggest that invasive and native populations had different specificity of elicitation to biotic and abiotic stimuli, especially, only either *S. litura* or *A. hygrophila* damage treatment expressed this specificity in invasive populations rather than both in native populations. However, the difference in herbivore performances did not vary between invasive and native populations. Documenting variation in specific plant induced response among populations under different herbivore pressure, like that which exists between the native and invasive populations, is one way to improve our understanding of the evolution of specificity of induced response (Bingham & Agrawal, 2010; Carrillo, McDermott, & Siemann, 2014). Future work may consider variation of specificity of induced response between invasive and native populations to herbivores with close phylogenetic relationship but different diet breadth.

## CONFLICT OF INTEREST

None declared.

## AUTHOR CONTRIBUTIONS

Mu Liu and Xiaoyuan Pan conceived the idea and designed the experiment, Mu Liu, Fang Zhou, and Zhijie Zhang performed the experiment, Mu Liu and Zhijie Zhang analyzed the data. Mu Liu and Xiaoyun Pan wrote the manuscript, all the authors contributed to revisions.
