# Supplementary material for: Specificity of herbivore‐induced responses in an invasive species, Alternanthera philoxeroides (alligator weed)
Source: Ecol Evol. 2017 Nov 23;8(1):59–70. doi: 10.1002/ece3.3615 (PMC5756832; doi:10.1002/ece3.3615)
Supplement: Supplementary file 1 [file ECE3-8-59-s001.docx]

| **Appendix S1** Sampling locations of *A. philoxeroides* | | | |  |
| --- | --- | --- | --- | --- |
| Collection site | Latitude | Longitude | Habitat | abundance of *A. hygrophila* |
| *Argentina (Native)* |  |  |  |  |
| Formosa | 25.46'S | 58.34'W | wetland | medium |
| Misiones | 27.15'S | 60.31'W | roadside | medium |
| Corrientes | 27.59'S | 56.20'W | roadside | medium |
| Santa Fe | 29.16'S | 59.49'W | river bank | low |
| Buenos Aires | 34.36'S | 58.37'W | pond side | medium |
| *USA (Introduced)* |  |  |  |  |
| Mississippi | 33.16'N | 88.47'W | river bank | none |
| Texas | 29.54'N | 93.57'W | wetland | none |
| Louisiana | 29.06'N | 90.08'W | wetland | none |
| Florida | 26.35'N | 81.30'W | river bank | none |
| Texas | 26.12'N | 97.38'W | wetland | none |
