# Supplementary figures and images for: Specificity of herbivore‐induced responses in an invasive species, Alternanthera philoxeroides (alligator weed)
Source: Ecol Evol. 2017 Nov 23;8(1):59–70. doi: 10.1002/ece3.3615 (PMC5756832; doi:10.1002/ece3.3615)

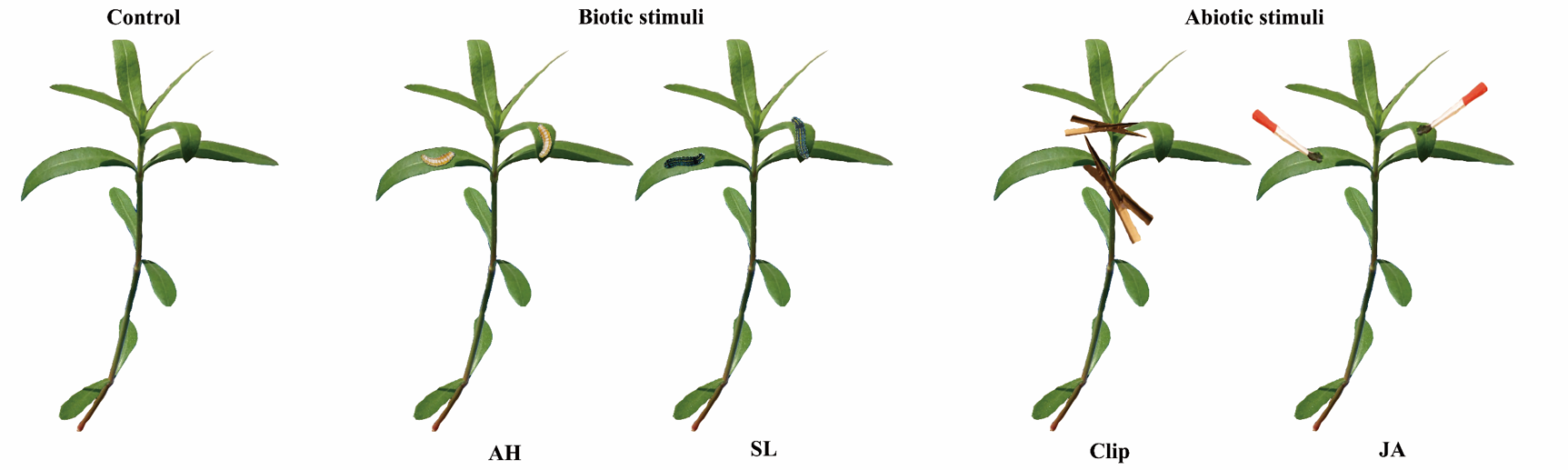

Supplement: Supplementary file 2 [file ECE3-8-59-s002.tiff]

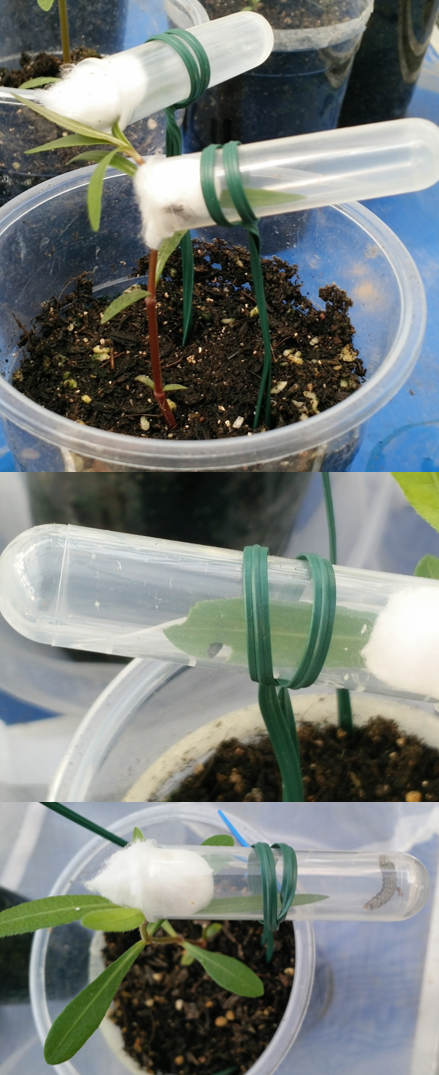

Supplement: Supplementary file 3 [file ECE3-8-59-s003.tiff]

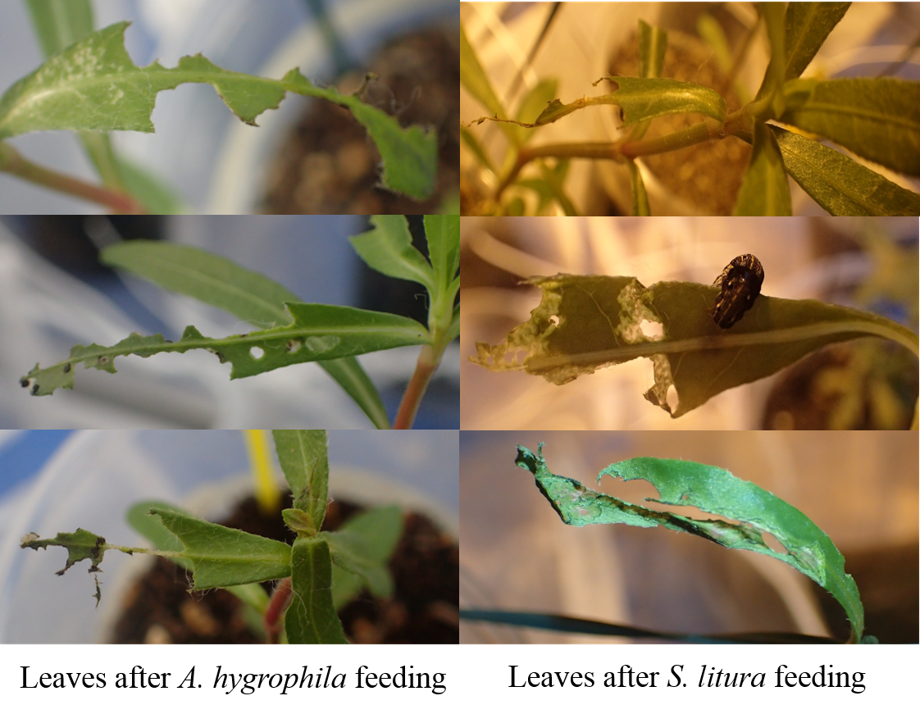

Supplement: Supplementary file 4 [file ECE3-8-59-s004.tiff]
